# Hydroxamate-based inhibitors reveal structural determinants of selectivity for *Plasmodium falciparum* aminopeptidase P

**DOI:** 10.1016/j.jbc.2026.111372

**Published:** 2026-03-17

**Authors:** Belinda J. Mills, Kyle S. Gregory, Gyles E. Cozier, Supannee Taweechai, Eve Loughlan, Glenn A. McConkey, R. Elwyn Isaac, Richard Foster, K. Ravi Acharya

**Affiliations:** 1 School of Chemistry, University of Leeds, Leeds, UK; 2 Department of Life Sciences, University of Bath, Bath, UK; 3 School of Biology, Faculty of Biological Sciences, Clarendon Way, University of Leeds, Leeds, UK

**Keywords:** malaria, *Plasmodium falciparum* aminopeptidase P, metalloprotease, apstatin, hydroxamic peptide inhibitor, X-ray crystallography

## Abstract

The malarial parasite, *Plasmodium falciparum (Pf)*, utilizes aminopeptidases in the breakdown of hemoglobin-derived oligopeptides to release amino acids for protein synthesis during growth and asexual reproduction of erythrocytic stages of the parasite. However, a N-terminal peptide bond that involves proline is difficult to hydrolyze. Aminopeptidase P (APP) is capable of cleaving peptide bonds with proline in the second position. Inhibition of *Pf*APP is therefore an attractive strategy for developing therapeutics for the treatment of malaria by limiting the supply of amino acids at the erythrocytic stage. We employed the structure-activity relationship of an existing APP inhibitor, apstatin, to design a more potent *Pf*APP inhibitor by introducing a hydroxamic acid metal-binding group in place of the amino-alcohol of apstatin and an aromatic P4′ moiety. A hydroxamic tetrapeptide with phenylalanine at P4′ (6d) greatly increased the inhibitory potency (apstatin *K*_i_, 16 μM; 6d, *K*_i_ 685 nM). Replacing the P3′ proline of 6d with a 2-substituted piperidine (6e) further improved the potency (*K*_i_, 24 nM). Crystal structure analysis of *Pf*APP in complex with 6d and 6e showed binding at the active site with coordination of the hydroxamic acid metal binding group to the di-metal center, and several protein–inhibitor interactions involving domains II and III. A comparison of *Pf*APP-6e with human APP1 indicated that the P4′ phenylalanine drives inhibitor potency and selectivity toward *Pf*APP, by forming an interaction with Tyr617 of the adjacent monomer within the dimer. The details presented here should be useful for the future design of potent and selective *Pf*APP inhibitors.

Malaria poses a major threat to approximately half of the world’s population. The emergence of resistance to front-line antimalarials in the most lethal human parasite species, *Plasmodium falciparum* (*Pf*), transmitted by blood-feeding *Anopheles* mosquitoes, is threatening the progress made in malaria control (1). The prospect of losing the efficacy of antimalarial drugs has provided impetus for the search of small molecule–based antimalarials with new modes of action. It has been established that asexual reproduction of the blood-stage parasite is critically dependent on the recycling of amino acids through catabolism of hemoglobin (Hb), which makes metalloaminopeptidases (MAPs), found in two key locations within the parasite, attractive targets for the development of new drugs. These aminopeptidases are responsible for the release of N-terminal amino acids from short peptide chains and were previously believed to reside and function only within the neutral environment of the cytoplasm (2). These MAPs contain one or two divalent metal ions within the active site, which are critical for catalytic activity (3) and exhibit unique substrate specificity profiles based on the nature of the active site binding pockets.

The *Pf* genome encodes eight MAPs, some of which have been found to be essential for parasite survival [for detailed reviews see (4, 5)]. In particular, two of these MAPs, aminopeptidase P (*Pf*APP) and alanyl aminopeptidase, have been reported to localize within a specialized acidic organelle, the food vacuole, and can act directly upon any short Hb-derived peptide fragments that meet their respective specificities (6, 7). The remaining peptide fragments are exported out of the food vacuole into the parasite’s cytoplasm where all eight MAPs are believed to work together to complete the degradation of the peptides to free amino acids.

*Pf*APP is a member of the M24 B subfamily of metallopeptidases responsible for the breakdown of specific peptides by catalyzing the removal of N-terminal residues in substrates containing a proline residue in the penultimate or so-called P1 position (8, 9). These peptide bonds incorporating the amino group of proline are not readily cleaved by aminopeptidases because of steric hinderance conferred by the cyclic imino group of the proline side chain. There are two classes of peptidases within the M24 B subfamily: prolidase and X-prolyl APP (9). Prolidases are responsible for the breakdown of Xaa-Pro dipeptides (where Xaa = any amino acid), whereas APP enzymes are responsible for the hydrolysis of the Xaa-Pro bond at the N terminus of proline containing oligopeptides. These APP enzymes have been found distributed across a wide range of organisms, including mammals and protozoa (10). There are three known isoforms of the enzyme in humans: APP1 (hAPP1, PDB ID: 3CTZ) (11) which is a soluble cytosolic form; hAPP2, a membrane bound form with an extracellular active site; and hAPP3 (PDB ID: 5X49) (12). hAPP3 has two isoforms of its own, one localized to the mitochondria and the other to the cytosol (11).

*P*. *falciparum (Pf)* possesses a single APP (*Pf*APP) that is a 157 kDa cytosolic protein that has been studied in detail and shown to be vital for the survival of the parasite (5, 6, 10). *Pf*APP is the only malaria parasite peptidase known to cleave Xaa-Pro oligopeptide substrates, thus supporting the critical role that the enzyme plays in Hb catabolism and parasite survival (5, 9).

The three-dimensional molecular structures of various APPs have been reported to good resolution and, in some cases, in conjunction with bound ligands (9, 10, 13, 14, 15, 16). Comparisons between the crystal structures of the enzyme from different species offers an insight into how the active site structures vary both across, and within, species and provides a molecular foundation for the rational development of APP inhibitors.

The most well-characterized selective inhibitor of APP is the peptidomimetic apstatin, which employs an amino-alcohol metal-binding group to coordinate the enzyme’s active-site divalent metal ions. However, apstatin exhibits limited inhibitory potency, highlighting the need to design new compounds with significantly higher affinity. Such advances are essential to enable the development of APP inhibitors with genuine therapeutic potential. Here we report the design and synthesis of two potent and novel hydroxamic acid-based *Pf*APP inhibitors, **6d** (*K*_i_ 0.685 μM) and **6e** (*K*_i_ 0.024 μM), developed through the structure activity relationship of a known *Pf*APP inhibitor, apstatin. We also present the crystal structures of *Pf*APP in complex with **6d** and **6e** (at 2.55 and 2.70 Å, respectively) and compare them with the previously reported structure of *Pf*APP in complex with apstatin (9). This combined set of results will now provide a sound basis for the further development of inhibitors of *Pf*APP of therapeutic value.

## Results

### Synthetic chemistry, kinetics, and biological activity of peptide-based hydroxamic inhibitors of PfAPP

**Figure 1:**
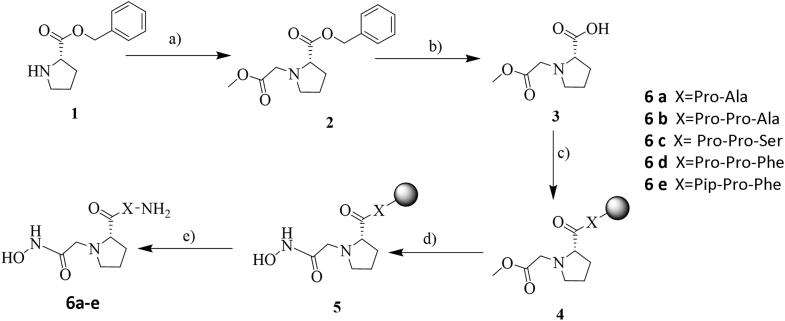
**Synthetic route for the synthesis of hydroxamic acid-containing peptides**. Reaction conditions: a) methyl chloroacetate, NEt_3_, THF, 88%; b) H_2_, 10% Pd/C, 98%; c) resin-bound peptide (X), DIC, Oxyma Pure, DMF; d) NH_2_OH.H_2_O (50% aq.), KOH (1M in MeOH), THF; e) TFA, H_2_O, TIPS. (for more details see experimental procedures section).

**Figure 2:**
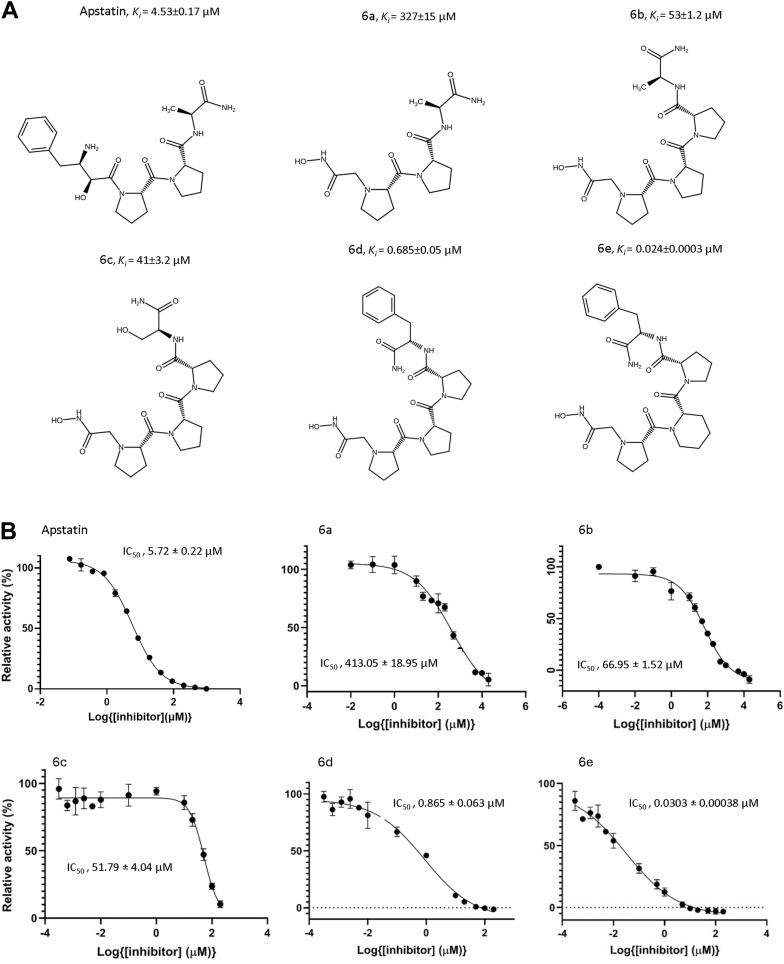
**Kinetic activity of novel *Pf*APP inhibitors**. *A*, molecular structures and inhibition (*K*_*i*_) data for apstatin and hydroxamic-peptide inhibitors, **6a**–**6e**. *K*i values were calculated from IC_50_ data using the Cheng–Prusoff equation (10, 20). *B*, plots for the inhibition of PfAPP by apstatin and hydroxamic-peptide inhibitors, **6a–6e**. Data are expressed as relative activity (%) *versus* increasing inhibitor concentration. Data are the mean values ± standard error of the mean of technical replicates (n = 4) and are plotted using a non-linear regression curve fitting program in GraphPad Prism. *Pf*APP, Plasmodium falciparum aminopeptidase P.

Apstatin is a peptide-based amino-alcohol that is a selective APP inhibitor but is a relatively weak inhibitor of *Pf*APP (10). To improve inhibitor potency, we used a discovery strategy based around designing analogs of apstatin with a different metal-binding group (MBG). Hydroxamic acids represent a significant class of inhibitors that have been successfully developed as inhibitors of metalloenzymes (17, 18). The hydroxamic acid MBG drives potency by forming strong interactions with enzyme active site metal ions. As a result of this, it was hypothesized that incorporating a hydroxamic acid MBG onto the tripeptide chain scaffold of apstatin might generate a more potent inhibitor of *Pf*APP. An initial synthetic route was designed to allow for the positioning of the hydroxamic acid MBG in relation to the first proline residue to best mimic the positioning of the metal-binding atoms in the apstatin amino-alcohol MBG (Fig. 1 and Fig. 2). First, N-alkylation of the benzyl-protected proline residue (**1**) was performed to yield ester **2**, followed by benzyl deprotection to reveal the free carboxylic acid **3**. The free carboxylic acid (**3**) was then coupled to a library of resin-bound peptide chains (X) using standard solid-phase peptide synthesis methods to produce the peptide-bound esters **4**. For the synthesis of **6e**, the ester **4** was converted into the corresponding hydroxamic acid **5** using aqueous hydroxylamine and methanolic KOH. Finally, hydroxamic acid **5** was cleaved from the solid support to yield **6e**. For synthesis of peptides **6a-d**, the peptide-bound esters **4** were cleaved from the resin to generate the esters **7** which were then converted using hydroxylamine and methanolic KOH to the peptide products, **6a-d**.

This scheme allowed for the efficient synthesis of a library of hydroxamic acid tripeptides and tetrapeptides that were then tested for their inhibitory potency against *Pf*APP (Fig. 2, *A* and *B*). Surprisingly, the initial replacement of the amino alcohol of apstatin with the hydroxamic acid MBG (**6a**) resulted in a decrease in inhibitor potency from a *K*_*i*_ of 4.53 μM to 327 μM, suggesting that the hydroxamic group may not be an effective MBG for the design of potent *Pf*APP inhibitors. Incorporation of an additional proline residue (**6b** and **6c**) increased the potency by ∼6- to 8-fold, but still these tetrapeptides remained less potent than apstatin. The introduction of a phenylalanine at the P4′ position of **6 b/6c** resulted in a tetrapeptide **6d** (*K*_i_, 0.685 ± 0.05 μM) that was over 6 times more potent than apstatin. Changing the P2′ residue of **6d** from a proline to the 6-membered ring analogue in **6e** increased *Pf*APP inhibitor potency even further, resulting in a *K*_i_ of 24 ± 0.3 nM.

Hydroxamic acid is a common MBG of inhibitors of various metalloproteases with different substrate specificities. In order to assess the selectivity of **6e,** the compound was tested for inhibitory activity toward other commercially available metalloproteases. These were human angiotensin converting enzyme, matrix metalloproteinase-1, neutral endopeptidase, and tumor necrosis factor-α converting enzyme. At a concentration of 10 μM, no significant inhibition was seen for **6e** against any of the metalloproteases tested (Table 1). Although the list of metallopeptidases is not exhaustive, these results are consistent with the predicted selectivity of this hydroxamic acid for APP.

**Table 1:** Effect of 10 μM 6e on the activity of a panel of human recombinant metalloproteases

Peptidase	Compound 6e
	% inhibition (10 μM)
Angiotensin converting enzyme (ACE)	−10
Matrix metalloproteinase-1 (MMP-1)	−2
Neutral endopeptidase (neprilysin)	−8
Tumor necrosis factor-α converting enzyme (TACE)	3

Assay methodology for testing the specificity of **6e** and **6d** using recombinant human enzymes carried out by Eurofins Panlabs Discovery Services Taiwan, Ltd. following established protocols (29, 30, 31, 32, 33, 34) (Supplementary Information Table S1). All substrates were added in 1.0% DMSO, and assays were performed in duplicate. Reference inhibitory compounds (captopril for ACE, GM-60001/galardin for MMP-1, phosphoramidon for neprilysin, GM-6001/galardin for TACE) were used to validate the assays.

**Figure 3:**
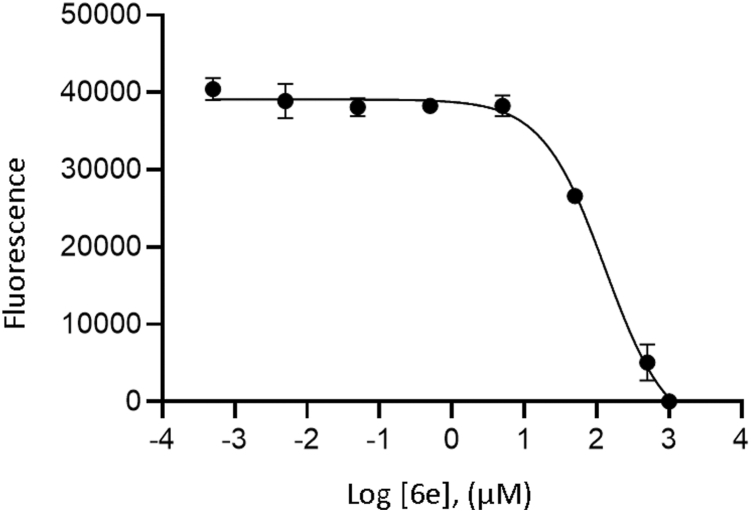
**Biological activity of hydroxamic acid inhibitor 6e.** Concentration-dependent inhibition of *Plasmodium falciparum* strain 3D7 growth by the hydroxamic acid inhibitor **6e**. Error bars, s.e.m., n = 4.

Since **6e** was the most potent inhibitor of *Pf*APP to date, it was tested for antiparasitic activity against the *P*. *falciparum* strain 3D7 in cultured red blood cells (Fig. 3). **6e** inhibited parasite growth with an IC_50_ of 163 ± 24 μM, whereas there was no effect of **6e** on a control human foreskin fibroblast line at a concentration of 500 μM.

### Absorption, distribution, metabolism, and excretion properties of compound 6e

As compound **6e** showed high potency toward recombinant *Pf*APP (Fig. 2*A*) but low potency using parasites in cell culture (Fig. 3), it was assessed for absorption, distribution, metabolism, and excretion profiling by Concept Life Sciences. The kinetic solubility and mouse liver microsomal stability, represented as both clearance (μL/min/mg) and half-life (min), were measured. In 0.1 M phosphate buffer pH 7.4, the kinetic solubility of **6e** was found to be relatively poor, but it possessed a low clearance level and relatively long half-life (Table 2) indicative of a low metabolic turnover *in vivo*. Furthermore, the permeability of **6e** was measured by Eurofins Scientific in the A-B permeability Caco-2 assay at pH 7.4 (19). **6e** performed poorly, with permeability measured at <0.033 x 10^-6^ cm/s. This result suggests that permeability is a significant issue and is a possible explanation for the lower potency of **6e** against parasite cultures compared to the inhibition of the recombinant enzyme. The nanomolar affinity of **6e** may compensate for its low permeability.

**Table 2:** ADME data of 6e collected by Concept Life Sciences

ADME properties	
Kinetic solubility	15.6 μM
Clint	6.2 μl/min/mg
Microsomal stability (mouse) (t_1/2_)	113 min

Kinetic solubility was measured in 0.1 M phosphate buffer at pH 7.4. Liver microsomal stability (mouse) is expressed as clearance in μL/min/mg and half-life in minutes. Experimental methodology is described in detail in the Supplementary Information section.

ADME, absorption, distribution, metabolism, and excretion.

### Crystal structures of PfAPP in complex with compounds 6d and 6e

To gain further insight into the structure activity relationship of the hydroxamic-peptide based *Pf*APP inhibitors, crystals of *Pf*APP in complex with **6d** and **6e** were grown by cocrystallization. The crystals belonged to the C2 space group, with two molecules (designated as molecule A and B) in the asymmetric unit (Table 3).

**Table 3:** X-ray data collection and refinement statistics of *Pf*APP in complex with compounds **6d** and **6e**

Crystallographic statistics		
	*Pf*APP-**6d**	*Pf*APP-**6e**
Resolution (Å)	98.57–2.55 [98.57–9.54] (2.65–2.55)	104.85–2.70 [104.85–9.73] (2.81–2.70)
Space group	C2	C2
Cell dimensions		
a,b,c (Å)	144.70,94.00,102.25	144.53,96.88,109.24
α,β,γ (°)	90.00,105.47,90.00	90.00,106.28,90.00
Number of molecules per ASU	2	2
Completeness (%)	100 [99.9] (100)	100 [99.9] (100)
R_Pim_	0.052 [0.009] (0.841)	0.091 [0.016] (0.611)
<I/σI>	10.1 [56.1] (1.1)	6.0 [31.3] (1.1)
CC_1/2_	0.999 [1.00] (0.525)	0.993 [0.999] (0.662)
Multiplicity	14.2 [13.7] (14.4)	13.9 [13.6] (14.3)
R_work_/R_free_	0.22/0.25	0.21/0.24
RMSD bonds (Å)	0.0084	0.0073
RMSD angles (°)	1.051	1.401
Ramachandran angles (%)		
*Favored*	96.5	94.97
Allowed	3.09	4.95
Outliers	0.41	0.08
Average B-factors (Å^2^)		
*Protein*	82	71
Ligand	70	54
Ions	68	63
Number of nonhydrogen atoms		
*Protein*	10,187	10,351
Ligand	76	78
Ions	4	4
PDB code	9T1X	9T1Y

The inner shell statistics are shown in square brackets, and the outer shell statistics are shown in parenthesis.

**Figure 4:**
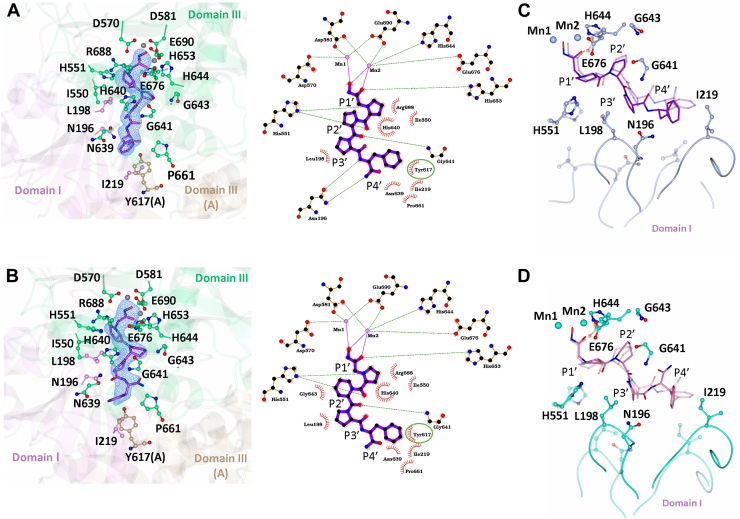
**Structure of *Pf*APP in complex with compound 6d (*Pf*APP-6d) and 6e (*Pf*APP-6e).***A*, mFo-Fc omit map electron density calculated in the absence of modeled ligand (contoured 3σ level) at the active site of *Pf*APP-**6d** molecule B (*left*) and Ligplot+ of interactions between *Pf*APP and **6d** (*right*). *B*, mFo-Fc omit map electron density calculated in the absence of modeled ligand (contoured 3σ level) at the active site of *Pf*APP-**6e** molecule B (*left*) and Ligplot+ of interactions between *Pf*APP and **6e** (*right*). Domain I is shown in *pink*, domain III in *green* (molecule B), and domain III (molecule A) in *brown*. Domain II does not interact with **6d** or **6e** and is omitted for clarity. Hydrogen-bond/electrostatic interactions are shown in *green*, and hydrophobic interactions are represented by *red semicircles*. The *Pf*APP residue that forms a π-stacking interaction is shown by the *green oval*. *C*, comparison of **6d** geometry and domain I dynamics in molecule A and B of *Pf*APP-**6d**. Molecule A is shown in transparent silver and Molecule B in silver. *D*, comparison of **6e** geometry and domain I dynamics in molecule A and B of *Pf*APP-**6e**. Molecule A is shown in *transparent teal* and Molecule B in teal. *Pf*APP, *Plasmodium falciparum* aminopeptidase P.

The structures were determined by molecular replacement using the apo *Pf*APP structure (PDB code 5JQK) to resolutions of 2.55 Å and 2.70 Å for *Pf*APP-**6d** and *Pf*APP-**6e**, respectively. The overall structure of both complexes are near identical (RMSD for Cα atoms of 1.4 Å and 1.1 Å, for *Pf*APP-**6d** and *Pf*APP-**6e**, respectively) to the native structure (9), which is comprised of a homodimer (monomer A and B). Each monomer consists of three domains: domains I (residues 121–304) and II (residues 305–475) are comprised of a seven stranded β-sheet core surrounded by five α-helices, whereas domain III (residues 476–777) contains the catalytic residues and forms the characteristic ‘pita-bread’ fold of the aminopeptidase family. *Pf*APP binds two manganese ions at the active site (Mn1 and Mn2), with Mn1 coordinated by residues Asp570, Asp581, Glu690, and Mn2 by Asp581, His644, Glu676, and Glu690. Inspection of the F_O_-F_C_ Fourier electron density map of *Pf*APP-**6d** and *Pf*APP-**6e** close to the Mn^2+^-binding sites revealed positive electron density for the entire inhibitor in both complexes (Fig. 4, *A* and *B*).

The coordination of the hydroxamic acid group to the di-metal center and most of the hydrogen bonding interactions are conserved between the two structures (Fig. 4, *A* and *B*). Residue His551 forms a hydrogen bond with the amine and carbonyl groups of the P1′ moiety in both structures. Additionally, Arg688, His551, His640, and Ile550 form a hydrophobic pocket occupied by P1′. Compounds **6d** and **6e** differ only at the P2′ position, with **6d** consisting of a 5-membered ring and **6e** a 6-membered ring, yet **6e** displays a ∼29-fold increase in *Pf*APP inhibition. There is a single hydrogen bond with the P2′ moiety, contributed by the amide nitrogen of Gly641, and this is present for both compounds. However, in the *Pf*APP-**6e** structure, the P2′ ring is positioned closer to Gly641, Gly643, and His644, increasing the number of potential van der Waals contacts **6e** can make relative to **6d** (Fig. 4, *C* and *D*). Except for Asn196, the residues that interact with the P3′ and P4′ moieties are conserved in both *Pf*APP-**6d** and *Pf*APP-**6e**, with Leu198 forming a hydrophobic contact with the P3′ proline and Asn639, Pro661, and Ile219 forming hydrophobic contacts with the P4′ phenylalanine. Interestingly, Tyr617 from the other molecule (molecule A) in the dimer also interacts with the P4′ moiety through a π-stacking interaction. In molecule B of *Pf*APP-**6d** only, there are two additional hydrogen bonds with the P4′ moiety (Fig. 4*A*). The interaction of **6d** with Asn196 is likely to be a weak, transient interaction, due to the flexibility of domain I. This is evidenced when we compare molecule A and B of both *Pf*APP-**6d** and *Pf*APP-**6e** (Fig. 4, *C* and *D*). In molecule A of *Pf*APP-**6d,** this interaction is absent, and Asn196 is positioned ∼7 Å from the P3′-P4′ peptide bond (Fig. 4*C*). In molecule B of *Pf*APP-**6e,** Asn196 is ∼4 Å from the P3′-P4′ peptide bond, whereas in molecule A, it is ∼8 Å away (as measured from the O^δ^ atom of Asn196 to the N atom of the P3′-P4′ peptide bond) (Fig. 4*D*). Furthermore, there is no electron density for the side chain of Ile219(A) of molecule A in *Pf*APP-**6d,** and in *Pf*APP-**6e,** Ile219(A) is positioned perpendicular to the P4′ phenylalanine moiety instead of parallel, with respect to molecule B. This movement of domain I, when comparing molecule B of *Pf*APP-**6d** (Fig. 4*C*) and *Pf*APP-**6e** (Fig. 4*D*) with molecule A, results in the loss of interactions with residues Asn196 (for *Pf*APP-**6d** only), Leu198, and Ile219. Given that **6e** is ∼29-fold more potent than **6d** and that the interaction with Asn196 was not observed for **6e**, this interaction does not appear to significantly improve *Pf*APP inhibitor potency.

### Comparison of 6d and 6e PfAPP complexes with the apo- and apstatin-bound PfAPP structures

**Figure 5:**
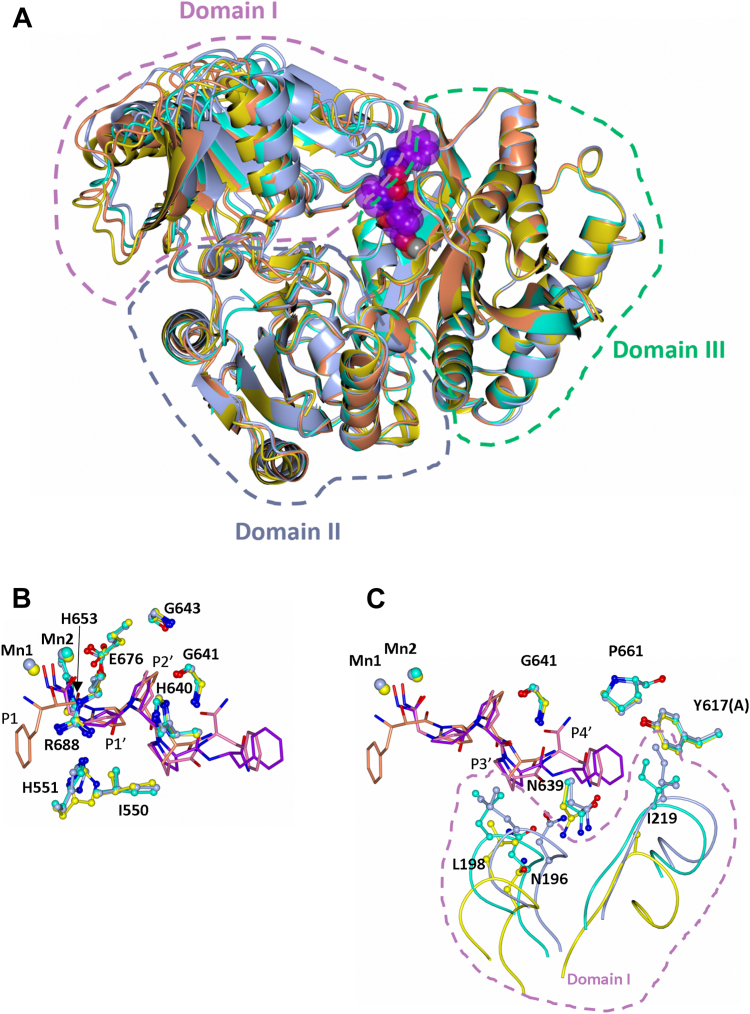
**Comparison of *Pf*APP-apstatin, *Pf*APP-6d, and *Pf*APP-6e**. *A*, superimposition of *Pf*APP-apo structure (molecule B, *orange*), *Pf*APP-apstatin (molecule A, *yellow*), *Pf*APP-**6d** (molecule B, *silver*), and *Pf*APP-**6e** (molecule B, *cyan*). **6e** is shown by the *purple spheres* to indicate the position of the active site. *B*, conformation differences between *Pf*APP-apstatin, *Pf*APP-**6d**, and *Pf*APP-**6e** relative to the P1′-P2′ positions. *C*, conformation differences between *Pf*APP-apstatin, *Pf*APP-**6d,** and *Pf*APP-**6e** relative to the P3′-P4′ positions. The amino acids of *Pf*APP-apstatin are shown in *yellow* and apstatin in *orange*, the amino acids of *Pf*APP-**6d** are shown in *silver* and **6d** in *purple*, and the amino acids of *Pf*APP-**6e** are shown in *cyan* and **6e** in *pink*. *Pf*APP, *Plasmodium falciparum* aminopeptidase P.

To determine the conformational changes that occur upon inhibitor binding, we compared the overall structures using only domain III to superimpose the *Pf*APP-apo structure (molecule B) with *Pf*APP-**6d** (molecule B), *Pf*APP-**6e** (molecule B), and *Pf*APP-apstatin (molecule A) (PDB code 5JR6) (Fig. 5*A*).

Across all four structures, the conformation of domain I is least similar, whereas domain II and III are near-identical (Table 4 and Fig. 5*A*) with only subtle changes to the position of amino acid sides chains.

**Table 4:** RMSD values for least squares minimization (LSQ) superimposition of domains I, II, and III of *Pf*APP-6d, *Pf*APP-6e, apo *Pf*APP (PDB code: 5JQK), and *Pf*APP-apstatin (PDB code: 5JR6)

Domain	6d-6e	6d-apo	6e-apo	6d-apstatin	6e-apstatin
I	1.36 Å	0.95 Å	0.91 Å	1.01 Å	1.02 Å
II	0.96 Å	0.74 Å	0.79 Å	0.86 Å	0.80 Å
III	0.60 Å	0.68 Å	0.61 Å	0.58 Å	0.62 Å

Values were calculated using coot (23), with residues 121 to 304 representing domain I, residues 305 to 475 representing domain II, and residues 476 to 777 representing domain III.

Furthermore, to fully investigate the dynamics of domain I in the available crystal structures, we compared molecule A and B of the asymmetric unit of each structure (data not shown). In all structures, domain I is tilted away from the active site in molecule A and toward it in molecule B (as seen in Fig. 2, *C* and *D* for *Pf*APP-**6d** and *Pf*APP-**6e**, respectively). This contributes to the differences in interactions observed between molecule A and B of the *Pf*APP-**6d** and *Pf*APP-**6e** structures and may explain why the previously determined *Pf*APP-apstatin structure, only showed binding in molecule A. As the tilt of domain I toward (molecule B) and away (molecule A) from the active site is seen in both the apo and holo-structures, crystallographic packing is the likely cause and would explain the transient interaction between **6d** and Asn196 described above. This does however reveal the dynamics of domain I which ‘caps’ the active site and may need to be dynamic to accommodate a range of substrates.

The interactions of **6d** and **6e**, compared to apstatin, at the di-metal center differ due to the presence of a hydroxamic acid group in place of the P1 phenylalanine moiety (Fig. 2*A*). The additional carbon preceding the P1′ amide group facilitates the interaction of the hydroxamic acid group closer to the di-metal center compared to the coordinating carbonyl and hydroxyl group of apstatin (Fig. 5*B*). Although **6d** and **6e** lack the presence of a bulky hydrophobic at the P1 position (Fig. 5*B*), the S1 pocket does not differ in conformation. The S1′ pocket shows flexibility with respect to His551, as in *Pf*APP-**6d** and *Pf*APP-**6e** His551 is positioned closer to the P1′ residue, compared to the *Pf*APP-apstatin structure (Fig. 5*B*). The relative proximity of the His551 to P1′ in *Pf*APP-**6d** and *Pf*APP-**6e** is indicative of its interaction with the ligand and absence in the *Pf*APP-apstatin structure. Furthermore, within the ASU of both *Pf*APP-**6d** and *Pf*APP-**6e,** there are subtle differences in the His551 position (Fig. 4, *C* and *D*). The P2′ moiety is identical in *Pf*APP-apstatin and *Pf*APP-**6d**; however, the P2′ carbonyl of *Pf*APP-apstatin does not interact with Gly641 as observed in the *Pf*APP-**6d** and PfAPP-**6e** structures (Fig. 5*B*). This is perhaps due to the increased flexibility of the of the P3′ alanine moiety of apstatin compared to the P3′ proline of **6d** and **6e** and may contribute to its reduced potency. The greater flexibility is likely due to the increased ϕ and ψ angle range that alanine can adopt, compared with proline, as well as the lack of an anchoring P4′ moiety. The interactions with the P3′ moiety for *Pf*APP-apstatin could not be determined due to low occupancy of the ligand. The position of residues within the putative S3′ and S4′ pocket, in particular those from domain I, vary between *Pf*APP-apstatin and *Pf*APP-**6d** and *Pf*APP-**6e** (Fig. 5*C*). This difference is likely due to the inherent flexibility of domain I within the crystal and indicates the interactions between domain I with **6d** and **6e** are transient/weak. The lack of a large P4′ in apstatin may explain its reduced potency for *Pf*APP as, unlike **6d** and **6e**, apstatin is not large enough to interact with both molecules of the dimer *via* a π-stacking interaction with Tyr617. This is further supported when we compare the *K*_*i*_ values of compounds **6b** and **6c** to **6d** (Fig. 2*A*), where introduction of the P4′ phenylalanine increased the affinity ∼77- and ∼60-fold, respectively.

### Comparison of PfAPP-6e and hAPP1 structures

**Figure 6:**
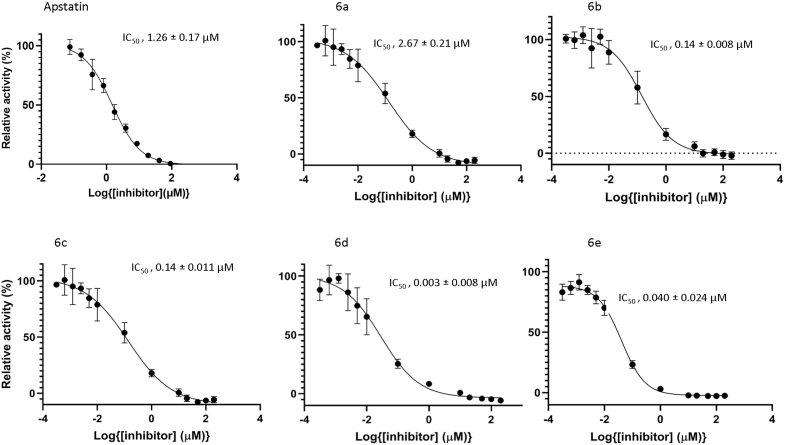
**Plots for the inhibition of hAPP by apstatin and hydroxamic-peptide inhibitors, 6a–6e**. Data are expressed as relative activity (%) *versus* increasing inhibitor concentration. Data are the mean values ± standard error of the mean of technical replicates (n = 4) and are plotted using a nonlinear regression curve fitting program in GraphPad Prism. hAPP, human aminopeptidase P.

As apstatin is an inhibitor of human APP, we further tested the affinity of the hydroxamic-peptide inhibitors **6a**, **6b**, **6c**, **6d**, and **6e** against hAPP1 (Fig. 6 and Table 5).

**Table 5:** Summary of *K*_*i*_ data

Peptidase	*K*_*i*_ (μM)					
	Apstatin	**6a**	**6 b**	**6c**	**6d**	**6e**
hAPP1	0.473 ± 0.065	1 ± 0.08	0.053 ± 0.003	0.053 ± 0.004	0.011 ± 0.003	0.015 ± 0.009
*Pf*APP	4.53 ± 0.17	327 ± 15	53 ± 1.2	41 ± 3.2	0.685 ± 0.05	0.024 ± 0.0003

Data were calculated by nonlinear regression on Graphpad prism, for hAPP1 and *Pf*APP with apstatin and its hydroxamic acid-based derivative: 6a, 6b, 6c, 6d, and 6e using the Cheng–Prusoff equation (20).

These data indicated nanomolar affinity for inhibitors 6b-e toward hAPP1. Interestingly, the introduction of the hydroxamic acid MBG onto apstatin (**6a**) resulted in a modest ∼2-fold decrease in the affinity for hAPP1which contrasted with the ∼72-fold reduction in inhibition seen with *Pf*APP. However, the additional modifications had less effect on potency of these compounds for hAPP1 compared to *Pf*APP. The P4′ Ala to Phe modification (**6b** to **6d**) had a ∼5-fold increase in potency for hAPP1, whereas for *Pf*APP, the increase was ∼77-fold. Furthermore, introduction of a piperidine ring at the P2′ position (**6d** to **6e**) further increased potency for *Pf*APP ∼29-fold, but for hAPP1, the potency decreased marginally.

**Figure 7:**
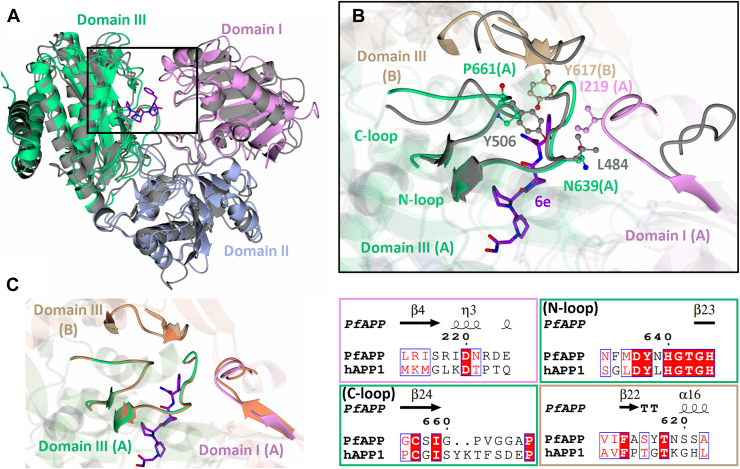
**Comparison of *Pf*APP-6e and hAPP1.***A*, superimposition of *Pf*APP-**6e** monomer with hAPP1 (PDB code 3CTZ), illustrating conservation of the core fold. *B*, comparison of domain I(A), domain III(A), and domain III(B) loops that make up the S4′ subsite in *Pf*APP with corresponding hAPP1 loops. A and B refer to separate *Pf*APP monomers within the dimer. *C*, superimposition of *Pf*APP-**6e** with Apo *Pf*APP structure (PDB code 5JQK) to illustrate minimal change within the S4′ subsite loops upon binding. Domains I, II, and III are shown in *pink*, *silver*, and *green*, respectively for *Pf*APP(A). For *Pf*APP(B), domain III is shown in *brown*. Apo *Pf*APP and hAPP1 are shown in *orange* and gray, respectively. Compound **6e** is shown in *purple*. The N- and C-loops of domain III(A) are indicated by ‘N-loop’ and ‘C-loop’, respectively. Figure was generated using CCP4mg and ESPript 3.0 (35). hAPP, human aminopeptidase P; *Pf*APP, *Plasmodium falciparum* aminopeptidase P.

To ascertain the molecular features for this greater increase in potency for *Pf*APP compared to hAPP1, we superimposed the crystal structure of *Pf*APP-**6e** with the native hAPP1 crystal structure (PDB code 3CTZ) (11). The structures aligned well with an RMSD value of 2.02 Å for 561 Cα atoms. The core fold of each of the domains is conserved but with differences in the loops between secondary structure (Fig. 7, *A* and *B*).

A comparison of the S2′ subsite indicates a single difference, where the *Pf*APP-Ser658 residue is replaced by hAPP1-Gly503; however, the Cβ atom of *Pf*APP-Ser658 is ∼6.3 Å away from the piperidine ring and therefore does not make direct interactions. The loops that make up the S4′ subsite in *Pf*APP differ in both structure and sequence compared to hAPP1 (Fig. 7*B*). The domain III(A) loops (N-loop and C-loop) are the only loops that structurally align between *Pf*APP and hAPP1, such that they likely contribute to formation of the S4′ subsite in both hAPP1 and *Pf*APP1. The structurally equivalent residue to *Pf*APP-Pro661 (of the C-loop) and *Pf*APP-Asn639 (of the N-loop) are hAPP1-Tyr506 and hAPP1-Leu484, respectively, which provide the potential for π-stacking and hydrophobic contacts with the P4′ Phe moiety of **6 d**/**6e** in hAPP1. These residues in the S4′ subsite of hAPP1 may explain the ∼5-fold increase in affinity between compounds **6 b**/**6c** and **6d**; however, hAPP1 lacks conservation of key residues in the domain I(A) and III(B) loops that contribute to binding of the P4′ moiety in *Pf*APP-**6e**, namely Ile219(A), and Tyr617(B). In fact, given that loop I(A) and III(B) are positioned away from the superposed ligand (Fig. 7*B*), they are unlikely to contribute to formation of the S4′ subsite in hAPP1. Although the conformation of these loops could change upon ligand binding, the domain I(A) and III(B) loops in hAPP1 include proline residues which restrict the flexibility of these loops, relative to *Pf*APP. Furthermore, a comparison of *Pf*APP-**6e** to the apo *Pf*APP structure (PDB code 5JQK) shows that all four loops adopt identical conformations in the bound and unbound structures (Fig. 7*C*), suggesting that these residues contribute to formation of the preformed active site in *Pf*APP and do not reposition on binding (induced fit). This is likely due to *Pf*APP-Ile219 of the domain I(A) loop positioning toward the active site to reduce contact with the bulk solvent, and the fact that the domain III(A) and domain III(B) loops form backbone hydrogen bonds with the core β-sheet fold of domain III, restricting their flexibility. In hAPP1, loops III(A) and III(B) also form backbone hydrogen bonds with the core β-sheet fold, suggesting they too are unlikely to adopt alternative conformation upon binding. This analysis, along with the presented kinetics, indicates that targeting of the S4′ subsite in *Pf*APP may be an effective strategy for the development of more potent and selective *Pf*APP inhibitors.

## Discussion

Apstatin is a small peptide-mimetic comprised a hydroxyl MBG and displays weak inhibition of *Pf*APP (*K*_i_, 4.53 μM). Introduction of a hydroxamic-acid MBG onto the tripeptide scaffold of apstatin (peptide sequence, PPA), a proline at the P2′ position, and a phenylalanine at the P4′ position, improved the *Pf*APP inhibition potency ∼7-fold (compound **6d** *K*_i_, 0.685 μM). Subsequent inclusion of a heterocyclic 6-membered ring at the P2′ position in place of the proline moiety of compound **6d** increased the affinity a further ∼29-fold (compound **6e** *K*_i_, 0.024 μM) resulting in a compound ∼189-fold more potent than apstatin for *PfAPP*. The crystal structures of the *Pf*APP dimer in complex with compounds **6d** and **6e** indicated clear binding *via* coordination of the hydroxamic acid to the two Mn^2+^ ions at the active site and several hydrogen bonds and hydrophobic contacts with domains I and III. In addition, Tyr617 from domain III of its dimeric partner was shown to form a π-stacking interaction with the P4′ phenylalanine moiety. This interaction is not feasible in apstatin, due to the lack of a P4′ moiety, and it appears to contribute significantly to the increased potency observed for **6d** and **6e** as introduction of the hydroxamic acid group alone (compound **6a**) decreased the potency ∼ 72-fold compared to apstatin. Furthermore, compounds **6b** and **6c**, which contain an alanine and serine as a P4′ moiety, respectively, also had a reduction in potency (Fig. 2*A*).

**Figure 8:**
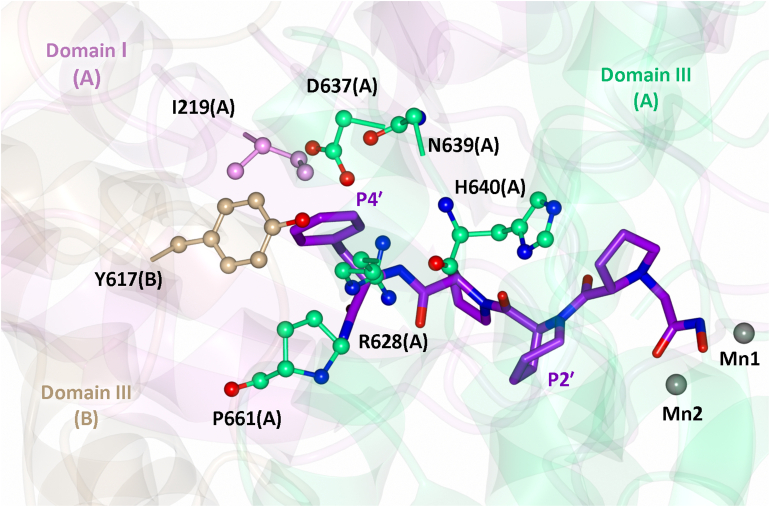
**The S4′ subsite of *Pf*APP**. The *Pf*APP-**6e** structure was used to represent the residues comprising the S4′ subsite, which is formed by domains I and III of one monomer (monomer A) and domain III of its dimeric partner (monomer B) in both molecules within the ASU. In *Pf*APP-**6d** and *Pf*APP-**6e**, the P4′ phenylalanine moiety occupies the S4′ subsite and forms a π-stacking interaction with Tyr617 which drives the increase in potency of **6d** and **6e** relative to apstatin. Domain I (A), III(A), and III(B) are shown in *pink*, *green*, and *brown*, respectively. Compound 6e is shown in *purple*. The Mn ions are shown by *gray spheres*. *Pf*APP, *Plasmodium falciparum* aminopeptidase P.

As **6e** displayed the highest affinity for *Pf*APP, the antiparasitic activity of **6e** was assessed by measuring *Pf* growth in human red blood cells. Compared with the *in vitro* activity, **6e** had reduced potency. Assessment of the absorption, distribution, metabolism, and excretion properties and permeability of **6e** indicates poor permeability likely contributes to this reduction. Future work could therefore be focused on assessing the impact of other MBGs or the inclusion of P1 moieties (Phe in apstatin) on *Pf*APP inhibition, with the goal of improving kinetic solubility and permeability, as initial introduction of the hydroxamic acid MBG decreased inhibitor potency. In addition, we show that the P4′ moiety contributes to the increased potency of compounds **6d** and **6e** against *Pf*APP. This suggests that the pharmacological properties of **6d**, **6e**, and their derivatives could be further optimized by exploring alternative substituents at the P4′ position, with the goal of engaging additional residues within the S4′ subsite (Fig. 8). This may be particularly important for achieving selectivity toward *Pf*APP over human APP, as the S4′ subsite exhibits sequence and structural variability between *Pf*APP and hAPP1 (Fig. 7). These differences likely contribute to the distinct gain in inhibitor potency observed, with a ∼77-fold increase for *Pf*APP compared to a ∼5-fold increase for hAPP1 upon modification from 6b to 6d (Fig. 2).

Future optimization efforts could focus on enhancing interactions with the *Pf*APP-specific residues, Tyr617 (Gly462 in hAPP1), Ile219 (Lys98 in hAPP1), and Pro661 (Tyr506 in hAPP1), by screening a range of aromatic/hydrophobic substituents to improve selectivity. Additionally, strategies may also include introducing polar/charged interactions with Asp637 (Asp482 in hAPP1) or Arg628 (Arg473 in hAPP1), as well as hydrogen bonding interactions with the backbone of His640 (His485 in hAPP1) to improve potency. Collectively, the structural and biochemical data presented should be helpful in the design of potent and selective *Pf*APP inhibitors with the goal of developing new treatments for malaria.

## Experimental procedures

### Reagents and solvents

Chemical reagents and solvents were attained from commercial suppliers, *e*.*g*.*,* Sigma Aldrich, and were not subject to any purification before use.

### Physical methods

Automated solid-phase peptide synthesis (SPPS) was conducted using a Liberty Blue Automated Microwave Peptide Synthesizer. Purification of compounds by automated RP chromatography was performed using a RP RediSep C18 column and was executed using a Biotage Isolera Flash Purification system with Spektra. Purification of compounds by manual RP chromatography was achieved using the Waters Sep-Pak C18 plus long cartridge (WAT023635). A step gradient of 0 to 100% MeOH + 0.1% formic acid in H_2_O + 0.1% in 10% increments was used whereby 0.5 to 1 ml of each increment was added to the column and collected in separate fractions.

*LC-MS*. LC-MS analysis was performed using a Thermo Scientific Dionex UltiMate with a gradient of MeCN (5–95%) in water, each containing 0.1% formic acid, with a flow rate of 1 ml min-1 on a short path C18 RP column. Compounds were detected using a diode array detector and a Bruker amazon speed mass spectrum analyzer.

*HPLC*. HPLC was performed using an Agilent 1290 Infinity HPLC system (Agilent), with a diode array detector. Chromatographic separations were performed using an InfinityLab Poroshell 120 EC-C18 (2.1 x 50 mm, 1.9 μm particle size, Agilent) or Supeclo C18 (2.1 × 50 mm i.d., 2.7 μm particle size; Supelco) column at 25 °C and using a mobile phase of water/acetonitrile with 0.1% trifluoroacetic acid with a gradient starting with 95% water and 5% acetonitrile and ending with 5% water and 95% acetonitrile at a flow rate of 0.5 ml/min. The diode array detector recorded the chromatogram at a wavelength of 254 nm.

*NMR*. NMR was performed using a two-channel Bruker AV3HD NMR spectrometer operating at 9.4 T (400 MHz 1H) and equipped with a 5 mm BBO probe or a two-channel Bruker AV-NEO NMR spectrometer operating at 11.7 T (500 MHz 1H) and equipped with a 5 mm DCH cryoprobe. Samples were prepared in the appropriate solvent, and the chemical shifts were reported in parts per million (ppm). Multiplicities are stated for each peak using the following abbreviations: s = singlet, bs = broad singlet, d = doublet, dd = doublet of doublets, dt = doublet of triplets, t = triplet, td = triplet of doublets, q = quartet, m = multiplet. Coupling constants (J) were measured in Hertz (Hz).

### General synthetic procedures

#### Procedure A: Automated solid-phase peptide synthesis

The coupling method for each peptide was run on a 0.25 mM scale with two deprotection cycles at 75 °C and two coupling cycles at 75 °C using *N*,*N*′-diisopropylcarbodiimide and Oxyma Pure. 4-methylbenzhydrylamine resin was used as the solid support

#### Procedure B: Manual SPPS method

4-methylbenzhydrylamine resin (0.3–0.8 mmol/g loading) was preswelled with DMF (∼5 ml) (0.25 mM) for 20 to 30 min. The carboxylic acid (5 eqv.), Oxyma Pure (5 eqv.), and *N*,*N*′-diisopropylcarbodiimide (10 eqv.) were dissolved in DMF (∼2 ml) and added to the resin and left to spin at room temperature for 3 h. After coupling, the terminal amino acid was deprotected using 20% piperidine/DMF (3 x 3 ml) for 20 min after each addition. Between each deprotection and coupling step, the resin was washed thoroughly using DMF (5 x 3 ml). Prior to each coupling step, the resin was preswelled with DMF (∼5 ml) for 20 to 30 min. After synthesis was complete, the resin was washed with DMF (3 x 5 ml), DCM (3 x 5 ml), and diethyl ether (2 x 5 ml) and left to dry under vacuum for 3 hours.

#### Procedure C: Treatment of the resin post-SPPS

Cleavage of the peptide from the resin was achieved using a cleavage cocktail of TFA/water/TIPS (95:3:2), unless otherwise stated. The resin and cleavage cocktail were spun for 2/3 hours, then the eluent was collected and blown off with N_2_, and the resulting oil stored at 4 °C until purification.

#### Procedure D: Automated RP purification

Purification was achieved using a linear gradient of 20 to 100% MeCN + 0.1% formic acid in H_2_O + 0.1% formic acid.

#### Procedure E: Manual RP purification

A Waters Sep-pak C18 column was primed with MeOH + 0.1% formic acid (10 ml) followed by H_2_O + 0.1% formic acid (10 ml). The compound being purified was dissolved in the minimum amount of MeOH + 0.1% formic acid and loaded onto the primed column. Elution was performed in 0.5 to 1 ml fractions using a linear gradient 0 to 100% MeOH + 0.1% formic acid in H_2_O + 0.1% formic acid in 10% increments.

#### Procedure F: Conversion of ester to hydroxamic acid

To the ester (1 eqv.) in THF, 50% aq. NH_2_OH.H_2_O (9 eqv.) was added followed by KOH (1M in MeOH, 2 eqv.). The reaction mixture was left to stir for 3 h, after which the solvent was removed under vacuum.

### Synthesis of hydroxamic acid peptides

#### Benzyl (2S)-1-(2-methoxy-2-oxoethyl)pyrrolidine-2-carboxylate **2**

H-Pro-OBz.HCl (3.00 g, 12.4 mM) was dissolved in dry THF (50 ml) and cooled to 0 °C, and NEt_3_ (5.23 ml, 37.2 mmol) was added. Methylchloroacetate (3.26 ml, 37.2 mmol) was added, the solution was stirred at 0 °C, and allowed to warm to room temperature overnight. The reaction mixture was diluted with H_2_O (50 ml) and extracted with EtOAc (5 x 50 ml). The combined organic extracts were washed with 1 M HCl (30 ml), sat. NaHCO_3_ (40 ml) and brine (30 ml) before being dried (MgSO_4_) and concentrated to leave the target compound as a colorless oil (3.03 g, 88%).

^1^H NMR δ_H_/ppm (400 MHz, CDCl_3_) 7.31 (5H, s, benzyl H), 5.11 (2H, t, *J* 15.2 Hz, benzyl CH_2_), 3.63 (3H, s, ester methyl H), 3.52 (2H, s, ethyl CH_2_), 3.13 (1H, t, *J* 8.3 Hz, pyrrolidine 5-C), 2.74 (1H, q, *J* 8.3 Hz, pyrrolidine 5-C), 2.14 (1H, q, *J* 8.1 Hz, pyrrolidine 3-C), and 1.70 to 2.02 (3H, m, pyrrolidine 3/4-C).

^13^ C NMR δ_C_/ppm (400 MHz, CDCl_3_) 173.6 (C=O), 171.2 (C=O), 135.9 (aryl 1-C), 128.5 (aryl 2-C), 128.2 (aryl 3-C), 128.1 (aryl 4-C), 66.3 (benzyl CH_2_), 63.7 (pyrrolidine 2-C), 53.5 (ethyl CH_2_), 53.0 (pyrrolidine 5-C), 51.4 (methyl C), 29.6 (pyrrolidine 3-C), and 23.8 (pyrrolidine 4-C).

#### (2S)-1-(2-Methoxy-2-oxoethyl)pyrrolidine-2-carboxylic acid **3**

Benzyl (2*S*)-1-(2-methoxy-2-oxoethyl)pyrrolidine-2-carboxylate (**2**) (3.03 g, 10.9 mmol) was dissolved in EtOH (20 ml) before 10% Pd/C catalyst (116 mg, 1.1 mmol) was added. The flask was evacuated under vacuum, and a balloon of hydrogen was attached. The solution was stirred at room temperature overnight. The reaction mixture was filtered through Celite to remove the catalyst, the Celite washed with EtOH (3 x 20 ml), and the filtrate concentrated under reduced pressure to afford the target compound as an orange oil (2.03 g, 98%).

^1^H NMR δ_H_/ppm (400 MHz, CDCl_3_) 5.81 (1H, br s, carboxylic acid OH), 3.69 (3H, s, methyl CH_3_), 3.51 to 3.57 (3H, m, ethyl CH_2_, pyrrolidine 2-H), 3.30 (1H, dt, *J* 8.1, 5.6 Hz, pyrrolidine 5-H), 2.70 (1H, dt, *J* 10.7, 5.6 Hz, pyrrolidine 5-H), 2.17 to 2.28 (1H, m, pyrrolidine 3-H), 2.06 to 2.12 (1H, m, pyrrolidine 3-H), 1.84 to 1.92 (1H, m, pyrrolidine 4-H), and 1.71 to 1.80 (1H, m, pyrrolidine 4-H).

^13^ C NMR δ_C_/ppm (400 MHz, CDCl_3_) 174.9 (C=O), 170.6 (C=O), 66.4 (ethyl CH_2_), 55.0 (pyrrolidine 2-H), 54.5 (pyrrolidine 5-H), 52.2 (methyl C), 30.7 (pyrrolidine 3-H), and 25.1 (pyrrolidine 4-H).

#### 2-{(2S)-N-[(2S)-1-Amino-1-oxopropan-2-yl]-2-[(2S)-pyrrolidine-2- carbonyl]pyrrolidin-2-yl}-N-hydroxyacetamide **6a**

The resin-bound dipeptide Pro-Ala was synthesized from the requisite Fmoc-protected amino acids following general procedure A and then (2S)-1-(2-methoxy-2-oxoethyl)pyrrolidine-2-carboxylic acid **3** installed using general procedure B. The peptide was cleaved from the resin using general procedure C (spinning for 2 h) with a cleavage cocktail of TFA:water (98:2). The crude was then purified following general procedure E to give methyl {(2S)-N-[(2S)-1-amino-1-oxopropan-2-yl]-2-[(2S)-pyrrolidine-2- carbonyl]pyrrolidin-2-yl}acetate as a colorless solid (LC-MS found: [M + H]+ = 355.37, 0.3 min; [M + H]+ requires 355.1981. HR-MS found: [M + H]+ = 355.1992; [M + H]+ requires 355.1981. The ester (181 mg, 0.51 mmol) was converted to the hydroxamic acid using general procedure F and the crude purified following general procedure D to yield the title compound as a yellow oil (173 mg, 95%). LC-MS found: [M + H]+ = 356.12, 0.4 min; [M + H]+ requires 356.1934. HRMS found: [M + H]+ = 356.1930 m/z; [M + H]+ requires 356.1934 m/z.

#### 1-{(2S)-N-[(2S)-1-Amino-1-oxopropan-2-yl]-2-[(2S)-pyrrolidine-2-carbonyl]-2- [(2S)-pyrrolidine-2-carbonyl]pyrrolidin-2-yl}-N-hydroxyacetamide **6b**

The resin-bound tripeptide Pro-Pro-Ala was synthesized from the appropriate Fmoc-protected amino acids following general procedure A, and then (2S)-1-(2-methoxy-2-oxoethyl)pyrrolidine-2-carboxylic acid **3** installed using general procedure B. The peptide was cleaved from the resin using general procedure C using TFA:water (98:2) as cleavage cocktail. The crude was dissolved in MeOH and 0.1% HCOOH (0.30 ml) and purified following general procedure E to yield the ester as a yellow solid. LC-MS found: [M + H]+ = 452.46, 0.4 min; [M + H]+ requires 452.2509. HR-MS found: [M + H]+ = 452.2333; [M + H]+ requires 452.2509. The ester (246 mg, 0.54 mmol) was converted into the hydroxamic following general procedure F. Purification was achieved following general procedure D to isolate the title compound as a yellow solid (241 mg). LC-MS found: [M + H]+ = 453.27, 0.4 min; [M + H]+ requires 453.2462. HR-MS found: [M + H]+ = 453.2459; [M + H]+ requires 453.2462.

#### 1-(N-Hydroxyacetamide)-Pro-Pro-Pro-Ser-NH2 **6c**

Resin bound Pro-Pro-Ser was synthesized from the requisite Fmoc-protected amino acids (using *t*-Bu protected Ser) following general procedure A and then (2S)-1-(2-methoxy-2-oxoethyl)pyrrolidine-2-carboxylic acid **3** installed using general procedure B. The resulting peptide was cleaved from the resin using general procedure C to yield the ester as a yellow oil. LC-MS found: [M + H]+ = 468.46, [M + H]+ requires 468.25). The ester (165 mg, 0.35 mmol) was then converted to the hydroxamic acid following general procedure F. The crude product was dissolved in MeOH + 0.1% HCOOH (0.6 ml) and purified following general procedure E and the title compound isolated as a yellow/orange oil (35 mg). HRMS found: [M + H] + = 469.2404 m/z; [M + H] + requires 469.2405 m/z.

#### 1-(N-Hydroxyacetamide)-Pro-Pro-Pro-Phe-NH2 **6d**

The resin-bound tripeptide Pro-Pro-Phe was synthesized following general procedure A and then (2S)-1-(2-methoxy-2-oxoethyl)pyrrolidine-2-carboxylic acid 3 was installed using general procedure B (leaving this final coupling reaction overnight). The resulting peptide was cleaved from the resin following general procedure C to yield the ester as an oil (LC-MS found: [M + H]^+^ = 528.56, [M + H]^+^ requires 528.28). The ester (137.2 mg, 0.26 mmol) was converted to the corresponding hydroxamic acid using general procedure F, and the crude dissolved in MeOH + 0.1% HCOOH (0.7 ml) and purified following general procedure E to give the title compound as a white solid (12.9 mg).

LC-MS found: [M + H]^+^ = 529.53; [M + H]^+^ requires 529.28. HRMS found: [M + H]^+^ = 529.2780 m/z; [M + H]^+^ requires 529.2769 m/z.

#### (2S)-2-{[(2S)-1-[(2S)-1-[(2S)-1-[(Hydroxycarbamoyl)methyl]pyrroidine-2-carbonyl]piperidine-2-carbonyl]pyrrolidin-2-yl]formamido}-3-phenylpropanamide , *A* and *B*

The requisite Fmoc protected amino acids and 2S)-1-(2-methoxy-2-oxoethyl)pyrrolidine-2-carboxylic acid **3** (3 eqv.) were coupled in sequence using general procedure B. The ester was converted to the hydroxamic acid using general procedure F, and the resulting peptide cleaved from the resin using general procedure C, dissolved in MeOH + 0.1% HCOOH (0.5 ml) and purified following general procedure E to give the. title compound as a yellow oil.

LC-MS found: [M+ H]^+^ = 543.10; [M+ H]^+^ requires 543.2931. HRMS found: [M+ H]^+^ = 543.2933 m/z; [M+ H]^+^ requires 543.2931.

### Protein production

The gene encoding the mature form of *Pf*APP (residues 121–777) was synthesized by Genewiz and amplified by polymerase chain reaction at 62 °C. Following extraction and gel purification, DNA was cloned into a pOPINF (His-tag) vector for expression. The His-tagged *Pf*APP gene was then transformed into BL21(DE3) *Escherichia coli* cells; the cells were grown at 37 °C in 2 L LB and induced with isopropyl β-D-1-thiogalactopyranoside overnight at 18 °C, followed by centrifugation; the resulting pellets were stored at −20 °C until required for protein purification.

His-tagged *Pf*APP was purified using a nickel affinity HisTrap HP column and run on an AKTA Pure chromatography system. Crude *Pf*APP was eluted from the column in the elution buffer (50 mM Tris, 300 mM NaCl, 400 mM imidazole, 5% glycerol, pH 7.6). The concentrated fractions containing protein were then injected into a Thermo Slide-A-Lyzer Dialysis Cassette and suspended in low salt buffer (50 mM Tris, 300 mM NaCl, 20 mM imidazole, 5% glycerol, pH 7.6) overnight followed by purification on a GE HisTrap FF purification column run on an AKTA Pure chromatography system using the elution buffer. The protein was further purified by size-exclusion chromatography using the enzyme storage buffer (12.5 mM Tris, 75 mM NaCl, pH 7.5). Fractions from the major peak, corresponding to dimeric *Pf*APP, were combined, concentrated to 3 mg/ml and stored at −80 °C.

### Inhibition assays

*Pf*APP was prepared and concentrated to 3 mg/ml. A stock solution of 50 μg/ml *Pf*APP was prepared by dilution with the enzyme storage buffer (12.5 mM Tris-HCl pH 7.5, 75 mM NaCl), aliquoted into 5 and 10 μl and stored at −80 °C until required. Stock solutions of the APP substrate, H-Lys(ABZ)-Pro-Pro-pNa (10 mM, Cambridge Bioscience) and the inhibitor, apstatin (50 mM, Insight Biotechnology) were prepared in DMSO and stored at −20 °C. The assay buffer used in all *Pf*APP assays, and for the dilution of reagents to use in the assays, consisted of 50 mM Tris-HCl, 250 mM NaCl, and 1 mM MnCl_2_, pH 7.5, and was prepared fresh on the day of the assay from stock reagents. Reaction rates were recorded using Greiner 384-well, PS, solid F-bottom, black plates (Merck Life Sciences), and a PerkinElmer EnVision 2103 Multilabel fluorescence plate reader, with the excitation wavelength set at 320 nm and the emission wavelength at 430 nm and the internal temperature control set at 37 °C. The assay comprised *Pf*APP (0.625 μg/ml, 10 μl), fluorogenic substrate (125 μM, 10 μl), and inhibitor (5 μl) and was conducted in quadruplicate. These components, apart from the fluorogenic substrate, were added to the wells of the assay plate which was incubated on ice for 1h before starting the reaction by the addition of substrate. The production of fluorescence from the hydrolysis of H-Lys(ABZ)-Pro-Pro-pNa was recorded continuously for 1h to determine the rate of reaction in the absence and presence of inhibitors. The % inhibition recorded for different concentrations of inhibitor was then analyzed using GraphPad Prism and plotted as a nonlinear regression curve with variable slope to generate IC_50_ values. The IC_50_ value was then converted to the *K*_i_ using the Cheng–Prusoff equation (20).

The testing of **6e** on the activity of human recombinant metalloproteases (angiotensin converting enzyme, matrix metalloproteinase-1, neutral endopeptidase, and tumor necrosis factor-α converting enzyme) was performed by Eurofins Panlabs Discovery Services Taiwan, Ltd. Enzyme assay details are presented in the Table S1.

### Crystallization and structure determination

*Pf*APP was concentrated to 8 mg/ml in a buffer containing 50 mM Hepes, pH 7.5, and 100 mM NaCl. Both **6 d** and **6e** compounds were dissolved to a concentration of 10 mM in water. *Pf*APP-**6 d** and *Pf*APP-**6e** complexes were prepared by mixing a 4:1 ratio of *Pf*APP and **6d**/**6e**, respectively. The samples were incubated at room temperature for 1 h prior to setting up of crystallization. Crystallization was performed by sitting drop vapor diffusion in 96-well plates using the Art Robbins phoenix crystallization nanodispenser and the high throughput BCS crystallization screen from molecular dimensions (Rotherham). Drops were screened in a 1:1 and 2:1 protein to reservoir ratio and incubated at 16 °C. The best crystals of *Pf*APP-**6d** grew in the 1:1 protein to reservoir drop containing 0.1 M Hepes, pH 7.5, 25% v/v PEG smear medium (12.5% w/v PEG 3350, 12.5% w/v PEG 4000, 12.5% w/v PEG 2000, 12.5% w/v PEG 5000 MME), and *Pf*APP-**6e** in 2:1 protein to reservoir drop containing 0.1 M Tris pH 8.5, 22% v/v PEG smear broad (4.55% v/v PEG 400, 4.55% v/v PEG 500 MME, 4.55% v/v PEG 600, 4.55% w/v PEG 1000, 4.55% w/v PEG 2000, 4.55% w/v PEG 3350, 4.55% w/v PEG 4000, 4.55% w/v PEG 5000 MME, 4.55% w/v PEG 6000, 4.55% w/v PEG 8000, and 4.55% w/v PEG 10000). Crystals were mounted using a litholoop and flash frozen in liquid nitrogen for data collection.

X-ray diffraction data were collected on beamlines I04 and I24, for *Pf*APP-**6d** and *Pf*APP-**6e** at Diamond Light Source (Didcot). Each crystal was kept at a constant temperature (100 K) using a liquid nitrogen stream and a total of 3600 images, at 0.1 ° of oscillation with exposure times of 0.01 s, were collected. Raw images were indexed and integrated with DIALS (21) and initial phases estimated by molecular replacement as part of the CCP4 suite (22). The phases were estimated using the *Pf*APP-apo structure (PDB code 5JQK). Refinement was performed using coot (23) and REFMAC5 (24). The Mn^2+^ ions, **6d,** and **6e** were modeled into the Fo-Fc Fourier electron density map and refined. A restraints dictionary for **6d** and **6e** were generated using AceDRG (25). All structures were validated with Molprobity (26), and figures were generated using CCP4MG (27). Atomic coordinates and reflection files were deposited to the protein data bank (PDB) under accession codes 9T1X and 9T1Y for *Pf*APP-**6d** and *Pf*APP-**6e,** respectively.

### Antiparasitic activity (IC50) determination

*P*. *falciparum* 3D7 parasites underwent two synchronization cycles with D-sorbitol before the day of assay. Human red blood cells (O+ blood was obtained from the National Blood service of the National Health Service Blood and Transplant Unit, Seacroft, Leeds) were washed with RPMI1640 medium by centrifugation at 3750 x g for 10 min. On the day of assay, the parasitemia and hematocrit were determined and adjusted to 0.5% and 3%, respectively. Next, synchronized ring parasites were grown in black-sided, clear flat-bottomed 96-well plates (Costar) in complete RPMI medium (with L-glutamine, 4-[2-hydroxyethyl]-1-piperazineethanesulfonic acid [Hepes], and phenol red; Thermo Scientific) supplemented with 5 g/L Albumax II (Gibco), 2 g/L sodium bicarbonate (Sigma-Aldrich), 0.1 g/L hypoxanthine (Sigma-Aldrich), and 0.1% (v/v) gentamicin (10 mg/ml, Gibco) with different inhibitor concentrations. A stock inhibitor solution (100 mM) was prepared in DMSO (Thermo Fisher Scientific). The dose–response tested inhibitor concentrations that varied 10-fold with serial dilutions from the highest concentration (eight total dilutions) added to 96-well plates. Wells without inhibitors were also prepared to represent maximum growth. Wells only containing uninfected red cells (3% hematocrit) in complete RPMI medium were prepared as negative controls for the assay background. Each compound was tested in triplicate.

Culture plates were incubated in 1% oxygen, 3% carbon dioxide, and 96% nitrogen in a humidified chamber at 37 °C. The chamber was re-gassed after 24 h, and plates were harvested after 48 h of incubation. Next, the relative amounts of live parasites were quantified using a fluorescence-based assay described by Smilkstein *et al*. (28). The technique uses the SYBR Green dye as a fluorescent marker for the parasites’ DNA. A 100 μl volume of 3 × lysis buffer was added to each well of the culture plates. The 3 × lysis buffer contained 0.024% (w/v) saponin (BDH), 0.24% (v/v) Triton X-100 (Sigma-Aldrich), 60 mM Tris (pH 7.5), 15 mM ethylenediaminetetraacetic acid, and 0.3 μl/ml of 1000 × SYBR Green I (Thermo Fisher Scientific). SYBR Green dye was added to the lysis buffer before use. After adding the lysis buffer, plates were incubated at room temperature for 45 min and protected from the light by covering them with aluminum foil. Fluorescence was measured with a multifunctional microplate reader (POLARstar OPTIMA, BMG Labtech) with excitation at 485 nm and detection at 520 nm.

The dose–response was determined by subtracting the background fluorescence of uninfected red cells from the fluorescence of each well before analysis using GraphPad Prism 9. The dose–response (parasites left over after 48 h of incubation) of each compound that showed growth inhibition was then recalculated by subtracting the fluorescence of the highest concentration from that of each measured concentration. Calibrated fluorescence values were plotted against the logarithm of the inhibitor concentration to determine the inhibitor’s IC_50_ value by a nonlinear regression curve fitting in the Prism software.

### Microsomal stability and kinetic solubility

Assays were conducted by Concept Life Sciences Ltd. (see Supplementary Information for details).

## Data availability

The X-ray diffraction reflection file and coordinates for *Pf*APP-6d and *Pf*APP-6e have been deposited to the RCSB protein data bank (www.pdb.org) under accession codes 9T1X and 9T1Y, respectively.

## Supporting information

This article contains supporting information.

## Conflict of interest

The authors declare that they have no conflicts of interest with the contents of this article.
