# Supplementary material for: Hydroxamate-based inhibitors reveal structural determinants of selectivity for Plasmodium falciparum aminopeptidase P
Source: J Biol Chem. 2026 Mar 17;302(5):111372. doi: 10.1016/j.jbc.2026.111372 (PMC13100289; doi:10.1016/j.jbc.2026.111372)
Supplement: Supplementary Material [file mmc1.docx]

**Supplementary information**

**Hydroxamate-based inhibitors reveal structural determinants of selectivity for *Plasmodium falciparum* aminopeptidase P**

**Belinda J. Mills^1, *^, Kyle S. Gregory^2, *^, Gyles E. Cozier^2,*^, Supannee Taweechai^3^,**

**Glenn A. McConkey^3^, R. Elwyn Isaac^3^, Richard Foster^1^ and K. Ravi Acharya^2^**

*From the ^1^ School of Chemistry, University of Leeds, Leeds LS2 9JT, UK; ^2^University of Bath, Department of Life Sciences, Claverton Down, Bath BA2 7AY, UK and ^3^Institute of Integrative and Comparative Biology, Faculty of Biological Sciences, Clarendon Way, University of Leeds, Leeds LS2 9JT, UK*

**Running title**

*PfAPP* inhibition by hydroxamic-peptides

**Keywords**

Malaria; *Plasmodium falciparum* aminopeptidase P; metalloprotease; apstatin; hydroxamic peptide inhibitor; X-ray crystallography

**Footnotes** -

For correspondence: K. Ravi Acharya ([bsskra@bath.ac.uk](mailto:bsskra@bath.ac.uk)); Richard Foster ([R.Foster@leeds.ac.uk](mailto:R.Foster@leeds.ac.uk)) and R. Elwyn Isaac ([R.E.Isaac@leeds.ac.uk](mailto:R.E.Isaac@leeds.ac.uk))

*These authors contributed equally to this work.

***Microsomal stability and kinetic solubility***

Metabolic stability assays were conducted in a 96-well plate format. Test compounds were incubated at a final concentration of 1 µM in a shaking incubator maintained at 37 °C. Mouse liver microsomes were diluted to 1 mg/mL and pre-equilibrated for 10 minutes at 37 °C in the presence of the 1mM NADPH cofactor. Biotransformation was initiated by addition of the test compound followed by mixing. Incubations were carried out with final solvent concentrations of 0.99% methanol and 0.01% DMSO. Aliquots (25 μL) were withdrawn at predetermined time points (0, 5, 10, 15, 25, and 35 minutes) and transferred into 300 μL of ice-cold methanol containing the internal standard (Tolbutamide). Reactions were terminated by mixing, and samples were centrifuged to precipitate proteins. Supernatants were analysed by LC-MS/MS using generic analytical methods to quantify the remaining parent compound at each time point. Intrinsic clearance and half-life values were calculated from the elimination rate constant obtained by plotting response ratio versus time.

***Kinetic solubility***

 Compounds were prepared as 10 mM DMSO stock solutions, which were thawed, sonicated, and 10µL spiked into 990 µL into 0.1M Phosphate buffer at pH 7.4. Samples were incubated in glass vials at 25 °C with shaking (850 rpm, 60 minutes) before centrifugation steps were performed to separate supernatant from undissolved solids. Supernatants were carefully transferred to clean vials and subjected to a second centrifugation. For analysis, aliquots of supernatants were prepared in 96-well plates with internal standard (Tolbutamide) to generate two sample dilutions (A and B). Standard curves were prepared from serial dilutions of DMSO stock solutions across a concentration range of 1.25 nM to 1.25 µM. All samples and standards were diluted in 30% methanol/70% water, mixed, and transferred to analysis plates for LC-MS/MS. Data processing included peak integration at consistent retention times, weighted curve fitting (linear or quadratic, 1/X or 1/X²), and exclusion of non-linear points as required. Appropriate dilutions were selected to ensure values fell within the standard curve range.

**Supplementary Table S1**. Assay conditions for testing the specificity of **6e** and **6d** using recombinant human enzymes carried out by Eurofins Panlabs Discovery Services Taiwan, Ltd. All substrates were added in 1.0 % DMSO and assays were performed in duplicate. Reference compounds were used to validate the assay for inhibition.

| **Enzyme** | **Enzyme source** | **Substrate** | **Buffer** | **Quantitation Method** | **Reference inhibitors** |
| --- | --- | --- | --- | --- | --- |
| Angiotensin Converting Enzyme (ACE) | Human recombinant Mouse myeloma cell line | 25.0 µM Abz-FRK (Dnp)-P | 50 mM MES, pH 6.5 | Spectrofluorimetric quantitation of Abz-Phe-Arg-OH | Captopril (IC_50_ , 2.24 nM) |
| Matrix Metalloproteinase-1 (MMP-1) | Human rheumatoid synovial fibroblast | µM Mca-Pro-Leu-Gly-Leu-Dap-AlaArg-NH_2_ | 50 mM MOPS, pH 7.2, 10 mM CaCl_2_, 10 µM ZnCl_2_, 0.05% Brij 35 | Spectrofluorimetric quantitation of Mca-Pro-Leu-Gly | GM-6001(IC_50_ , 17.8 nM) |
| Neutral Endopeptidase | Human recombinant CHO cells | 10.0 µM Mca-Arg-Pro-Ppro-Gly-Phe-Ser-Ala-Phe-Lys(Dnp)-OH | 50 mM Tris-HCl, pH 9.0, 0.05 % Brij-35 | Spectrophotometric quantitation of Mca-Arg-Pro-Pro-Gly-Phe-Ser-Ala | Phosphoramidon (IC_50_ , 1.99 nM) |
| Tumour Necrosis Factor-α Converting Enzyme (TACE) | Human recombinant insect Sf21 cells | 0.0 µM Mca-Pro-Leu-Ala-Gln-Ala-Val-Dpa- Arg-Ser-Ser-Ser-Arg-NH_2_ | 5 mM Tris-HCl, pH 9.0, 2.5 µM ZnCl_2_,  0.05% Brij 35 | Spectrophotometric quantitation of Mca-Pro-Leu-Ala-Gln | GM-6001 (IC_50_ , 12.7 nM) |
